# Reference values for electroglottographic data of elderly women

**DOI:** 10.1590/2317-1782/20232021244en

**Published:** 2023-11-10

**Authors:** Bárbara de Faria Morais Nogueira, Ana Cristina Côrtes Gama, Flávio Barbosa Nunes, Adriane Mesquita de Medeiros

**Affiliations:** 1 Departamento de Fonoaudiologia, Faculdade de Medicina, Universidade Federal de Minas Gerais - UFMG, Belo Horizonte (MG), Brasil.; 2 Programa de Pós-graduação em Ciências Fonoaudiológicas, Universidade Federal de Minas Gerais - UFMG - Belo Horizonte (MG), Brasil.; 3 Departamento de Oftalmologia e Otorrinolaringologia, Faculdade de Medicina, Universidade Federal de Minas Gerais - UFMG, Belo Horizonte (MG), Brasil.

**Keywords:** Elderly, Voice, Aging, Phonation, Women

## Abstract

**Purpose:**

To present reference values for fundamental frequency data and closed-phase quotient extracted from electroglottography in older women and verify if they differ from laryngeal examination results.

**Methods:**

Observational analytical cross-sectional study in 73 older women, aged 60 to 84 years, using electroglottographic and laryngeal examinations. Each participant’s voice sample had a sustained prolonged vowel /a/ emitted at the usual frequency and intensity, in the modal register. Fundamental frequency (F0) and vocal fold contact quotient (CQ) were extracted from the electroglottography. Data were descriptively analyzed with measures of central tendency and dispersion of continuous variables. To establish whether continuous electroglottography variables differed from the laryngeal diagnosis, Student’s t-test (parametric test) and Mann-Whitney (non-parametric test) were performed. The significance level in all tests was set at 5%.

**Results:**

Most participants did not have laryngeal changes, while 27.4% had presbylarynx. The mean electroglottographic measures were 187.24 Hz for F0 and 48.49% for CQ. There was no statistical significance between laryngeal examinations and EGG measures.

**Conclusion:**

Older women have mean electroglottographic F0 values of 187.24 Hz and CQ of 48.49%. Electroglottographic F0 and CQ measures did not change with the presence of the presbylarynx in older women.

## INTRODUCTION

The onset and progress of the natural aging of the voice depend on physical and mental health, individual life history, and constitutional, racial, hereditary, dietary, social, and environmental factors^(1)^. The first signs of aging occur around 60 years old, when individuals are chronologically considered older adults by the World Health Organization (WHO)^(2)^. Vocal changes in this period, known as presbyphonia, include reduced vocal intensity, difficulties in laryngeal muscle control, and respiratory limitations^(3-5)^. Changes in voice production resulting from this process may or may not be associated with presbylarynx^(5)^, which is the natural aging of the larynx due to advancing age.

Videolaryngoscopy of older adults with suggestive changes due to presbylarynx shows various modifications in the larynx, such as its lower position in the neck, glottic incompetence, atrophy and thinning of the vocal folds, arching and irregularity in vocal fold vibration, compensatory medialization of the ventricular bands, ossification and calcification of the cartilages, fragility of blood vessels with a tendency to submucosal hemorrhage, and prominence of the vocal process^(4-6)^. In the normal aging process, vocal fold atrophy is present in up to 60% of individuals after 60 years old, showing evidence of glottic insufficiency^(4)^.

Presbyphonia is diagnosed by exclusion^(5)^. Therefore, it is crucial to survey their medical history in detail and analyze the presence of laryngeal pathologies, comorbidities, systemic diseases, use of medications, and treatments that may be interfering with vocal quality. The multifactorial nature of dysphonia should be considered, and clinical reasoning should be based on what is expected for the diagnosis of presbyphonia.

Performing electroglottography (EGG) helps better understand laryngeal function, as it evaluates the closed phase of the glottic cycle. It allows for the identification of possible laryngeal alterations. EGG is an objective, non-invasive, simple measurement method, used to estimate vocal fold movement during phonation^(7)^. This method provides an indirect measure of vocal fold contact during phonation (vocal fold contact quotient - CQ), which shows the proportion of time the glottis remains closed relative to the total glottic cycle time^(8)^. This measure is obtained from the variation in electrical current, which changes as the vocal folds increase and decrease their degree of contact^(9)^.

With aging, changes are expected in electroglottographic measures due to the structural modifications that occur in the vocal folds as one gets older. CQ can be affected by changes in glottic closure, and the electroglottographic fundamental frequency (F0 EGG) can be influenced by anatomical and functional changes observed in the larynx of older individuals^(8,9)^. Understanding the EGG characteristics of CQ and F0 EGG measures among older people with and without presbylarynx is important to support the assessment of this age group, considering that the anatomical and functional differences in their larynx can impact the values of these measures. The literature presents reference values for EGG measures in adult^(10-14)^, pediatric^(15)^, and older populations^(8,16,17)^, but few studies have analyzed the behavior of EGG measures in Brazilian Portuguese speakers^(11-14)^.

Aspects related to speech tasks^(8)^ and linguistic variations resulting from cultural language patterns^(12)^ can interfere with the values of EGG measures. Therefore, analyzing EGG parameters in the older Brazilian population can help assess the vocal function in this age group and contribute to the use of objective vocal function measures in the multidimensional management of vocal disorders. This study aimed to answer the following questions: What are the values of EGG measures in older women? Do the results differ in the presence of presbylarynx? It is hypothesized that the anatomical and functional modifications of the vocal folds due to aging^(4-6)^ decrease CQ values (indicating a shorter closed phase of the glottic cycle) and increase F0 EGG.

Thus, this research aimed to present reference values for F0 and CQ data extracted from EGG in older women and determine if the results differ from laryngeal assessment findings.

## METHODS

This is a cross-sectional analytical observational study, approved by the Research Ethics Committee under protocol number 2,648,174.

All study participants signed an informed consent form and underwent the following procedures in the specified order: a questionnaire gathering data on age, educational attainment, retirement status, previous speech therapy treatment, and EGG evaluation conducted by one of the researchers to extract F0 EGG and CQ data. After the recording, the participants scheduled an otolaryngological evaluation (ORL) within 15 days. The ORL evaluation included a laryngoscopy performed by a single physician and the application of the translated version of the Reflux Finding Score (RFS) protocol^(18)^.

The inclusion criteria were being a woman aged 60 years or older capable of answering questions related to age, not having received speech therapy treatment for voice problems in the previous 12 months, and being able to easily perform the tasks requested for laryngeal and vocal assessment. The exclusion criteria for this study were an otolaryngological diagnosis of laryngeal changes that do not characterize presbylarynx, diagnosis of laryngopharyngeal reflux according to the RFS protocol, and failure to undergo laryngeal assessment.

The research took place at the OSF (Speech-Language Pathology Outpatient Clinic) of a public university, where EGG voice recordings were made. Videolaryngoscopy was performed by an otolaryngologist at a hospital affiliated with the same university.

Participants were sampled by convenience. The research was announced in community outreach programs with informative posters placed in the vicinity and corridors of the university and university hospital. The informative poster contained the study objective, inclusion and exclusion criteria, and the researcher’s contact email and phone number. The research team also approached people in person near the university hospital to disseminate information about the study and invite older women in the vicinity to participate. Those who were interested provided their name and contact phone number for subsequent communication and to schedule data collection appointments. It is worth noting that none of the older women contacted the research team through the community outreach poster. All participants in this research were recruited by in-person approach and were subsequently scheduled for data collection appointments by the researcher who contacted them by phone.

A total of 158 older women were invited to participate. All of them were scheduled, and 95 attended the OSF for data collection. Of those, only one older woman did not undergo an EGG examination, whereas 21 did not show up on the scheduled day for the laryngeal examination. Therefore, 22 participants were excluded from the study, resulting in a total of 73 older women participating in this study.

The voice material collected from each participant consisted of a sustained prolonged vowel /a/ emitted at their habitual frequency and intensity, in the modal register. All participants were instructed to produce the vowel at comfortable frequencies and intensities, and the speech-language pathologist perceptually monitored whether the productions were habitual. The participants were comfortably seated to minimize their movements during EGG waveform pick-up - to which two electrodes, cleaned and depolarized with saline solution, were symmetrically and superficially placed on the laminae of the thyroid cartilages at the level of the vocal folds, connected to and directly digitized on the computer. Data were collected in a quiet environment with ambient noise levels below 50 dB SPL, verified with a Radio Shack® sound pressure level meter and CSL program from Kay PentaxTM, model 6103, Lincoln Park, NJ, USA - EGG module installed on a Dell® computer, Optiplex GX260 model, with a professional sound card from Direct Sound®. The intensity was not objectively controlled during sustained vowel production.

The following EGG measures were extracted: F0 EGG, which is the number of glottic cycles produced in 1 second, measured in hertz (Hz); and CQ, which is the percentage (%) of time in each glottic cycle when the vocal folds are closed.

After collecting EGG measures, the participants underwent laryngeal evaluation, conducted by an otorhinolaryngologist using videolaryngoscopy. The procedure included a 70° telescope by Storz®, a 300-watt xenon light source by Storz®, and a telecam DX microcamera by Storz®️. These evaluations took place at least 15 days after EGG. All participants were instructed to sustain the vowel /i/ for a minimum of 2 seconds at their habitual frequency. The examination findings, including the RFS translated version^(18)^, were recorded in a medical report. At the end of the examination, all participants received a copy of their medical report, and the researcher received a copy of each participant's examination.

RFS evaluates subglottic edema (absent/present), ventricular obliteration (partial/complete), erythema or hyperemia (limited to the arytenoids/diffuse), vocal fold edema (mild/moderate/severe/polynomial), diffuse laryngeal edema, interarytenoid region hypertrophy (mild/moderate/severe/obstructive), granuloma/granulation tissue (absent/present), and thick endolaryngeal mucus (absent/present). The final score is obtained by summing all items on the protocol. A score above 7 indicates a diagnosis of RLF^(18)^.

The otorhinolaryngological diagnosis of presbylarynx was defined based on the presence of: 1) glottic gap; 2) atrophy and thinning of the vocal folds; and 3) prominence of the vocal process^(5)^. Laryngeal examination results were divided into two groups: no laryngeal changes and the presence of presbylarynx. The criteria for the group without laryngeal changes were the absence of mucosal changes, complete glottic closure of the vocal folds, and not meeting RFS criteria to diagnose RLF. To be classified in the presbylarynx group, the older women had to present all three aforementioned laryngeal signs^(5)^ and not meet RFS criteria to diagnose RLF.

After data collection, the information was digitized in a database using the Microsoft Office Excel program. All analyses were performed using IBM - SPSS Statistic Base software, version 25.0. Descriptive data analysis was conducted by analyzing the measures of central tendency and dispersion of continuous variables, and the Shapiro-Wilk test was used to assess normality. The comparison between groups with and without laryngeal changes regarding EGG measures was analyzed using the Student’s t-test (parametric) and Mann-Whitney test (non-parametric). The significance level was set at 5% for all tests.

## RESULTS

The research was conducted on 73 older women, with a mean age of 69 years (standard deviation = 5.75). Most participants had finished high school (31.5%) and were retired (90.4%). The participants’ mean CQ was 48.49 (standard deviation = 8.15) and the mean F0 was 187.24 Hz (standard deviation = 31.76). Laryngeal changes were absent in 72.6% of participants, while 27.4% had a presbylarynx. Presbylarynx was described in the laryngeal examination as vocal fold bowing with a fusiform gap. None of the study participants were diagnosed with RLF according to the laryngoscopy protocol used. The measures of central tendency for age and EGG measures are presented in Table 1.

**Table 1:** Descriptive measures of age, contact quotient, and fundamental frequency with electroglottography

**Variables**	**N**	**Mean**	**SD**	**Median**	**Minimum**	**Q_1_**	**Q_3_**	**Maximum**
Age (years)	73	69.22	5.75	69	60	65	73	84
Closed-phase quotient	73	48.49	8.15	48.16	6.9	43.81	52.62	68.7
Fundamental frequency	73	187.24	31.76	186.15	127.37	166.49	203.98	264.65

**Caption:** N= number of individuals; SD= standard deviation; Q= quartile

In Table 2, EGG measures (CQ and F0) were compared between the groups without laryngeal changes and with the presbylarynx, and no statistical differences were found between them.

**Table 2:** Comparison of contact quotient and fundamental frequency with electroglottography (n=73) between the groups

**Variables**	**Groups**		
	No laryngeal changes	Presbylarynx	**P-value**
	(n=53)	(n=20)	
**Fundamental frequency** **1** **(in Hz)**			
Mean	187.31	187.04	0.9745
Median	186.76	185.70	
**Closed-phase quotient** **2** **(in%)**			
Mean	47.41	49.39	0.3105
Median	47.87	49.57	

1 Student’s t-test;

2 Mann-Whitney test

**Caption:** Hz = Hertz

## DISCUSSION

Older women in this study had no different F0 EGG and CQ measures, based on laryngeal diagnoses (no changes vs. presence of presbylarynx). The low prevalence (27.4%) of presbylarynx found in this study may be ascribed to the participants age range. Half of the older women were below 69 years old, and only 25% were between 73 and 84 years old. A study with 104 healthy older women above 65 years old found a 45.2% prevalence of presbylarynx (fusiform gap)^(17)^.

There is a scarcity of studies defining normal parameters for EGG measures in older women^(8,16,17)^. The results of this research showed F0 EGG values of 187.31 Hz in older women without laryngeal changes and 187.04 Hz in those with presbylarynx. These results are similar to the values reported in the literature for older women, respectively 186.05 Hz^(8)^ and 211.06 Hz^(17)^.

Considering the population of adult women, mean F0 EGG values were reported as 226.91 Hz in female singers^(11)^, 204.87 Hz in adult women without vocal changes^(12)^, and 211.69 Hz in women without laryngeal changes^(14)^. The results suggest that F0 EGG values in the adult female population tend to be slightly higher than those found in older women.

We observed that the presence of presbylarynx in the older population does not impact F0 EGG values, as reported in the literature^(17)^. This suggests that the presence of laryngeal signs such as mucosal atrophy and vocal fold thinning^(5)^ does not significantly affect the F0 EGG parameter. In other words, age is a more important factor in determining F0 than the visible vocal fold changes identified in the laryngeal examination. It is reasonable to assume that aging leads to structural changes in the vocal folds^(5)^ that impact F0 EGG, regardless of whether these laryngeal changes are visually identified in laryngological evaluations.

It is important to note that extracting F0 as an EGG measure differs from obtaining it through acoustic soundwave analysis. F0 is easier to extract in EGG than in acoustic analysis because it represents clearer cycles^(1)^. Acoustic analysis obtains voice signals using some acoustic parameters, such as F0^(19)^, whereas EGG estimates the contact area between the vocal folds during voice production, from which F0 can also be derived^(1)^.

EGG data allows us to analyze the vocal fold contact during the glottic cycle, and it is used to assess vocal function^(7,10,13)^. The present results can help analyze EGG measures in older women extracted with the CSL program by Kay Pentax™️. However, it is important to note that differences in programs and evaluation criteria can alter EGG parameter results^(8)^ - hence, research findings should be cautiously compared.

The mean 48.49% CQ found in this study falls within the normal range (40-60%) described in the literature^(10)^, suggesting that aging does not interfere with this EGG parameter. In this research, older women without laryngeal changes had CQ values of 47.41%, while those with presbylarynx had CQ values of 49.39%, with no statistically significant differences between them. Glottic insufficiency has been demonstrated as a predominant characteristic of presbylarynx^(4)^. With advancing age, reduced glottic closure duration in women is expected^(16)^. However, the presence of a glottic gap, observed in all older women in the presbylarynx group, did not affect the CQ values. A study on CQ in 96 older women with and without presbylarynx found a mean value of 47.6%^(17)^. The CQ values were also inconclusive in identifying the presence of a glottic gap in older women, confirming the findings of this research. The authors discuss the possible interference of voice pitch and intensity in CQ evaluation^(17)^.

The results of this study suggest that aging has an impact, compared to the adult population, on F0 EGG values, with a slight reduction in these measures, but does not interfere with glottic closure time and, consequently, with CQ values, which are similar to those of the adult female population. These results are consistent even in the presence of presbylarynx. It is reasonable to assume that the anatomical and functional changes observed in the older population, especially in cases of presbylarynx, are not sufficient to modify EGG measures in this population. This suggests that anatomical and functional changes resulting from aging decrease the vibration speed of the vocal folds, leading to a decrease in F0 EGG, but do not change the duration of the closed phase of the glottic cycle, thereby not impacting CQ values. Standardization and prior experience with EGG are advisable, with better control over voice pitch and intensity during vocal emission.

The limitations of the study include the small sample size, particularly in the presbylarynx group, and the convenience sampling method, which prevents the generalization of the results. Additionally, the scarcity of scientific studies with EGG results in the older population and the lack of standardized measures make the interpretation of the results challenging.

This research helped identify reference values for F0 and CQ data extracted from EGG in different laryngeal conditions in the older population, aiding the multidimensional voice assessment of older women. Normative data are needed to analyze the conformity, reliability, and variability of vocal parameter measures across different age groups and sexes. Further studies should investigate the differences in CQ results with laryngeal characteristics (presbylarynx and normal larynges) among older women considering age groups.

Additional EGG studies in older individuals of both sexes are necessary to assess the possibility of standardizing this instrument in this population and its contribution to diagnosing presbylarynx and its vocal impacts.

## CONCLUSION

In older women, the mean F0 EGG value was found to be 187.24 Hz, and the mean CQ value was 48.49%. F0 EGG and CQ values did not show any significant changes with the presence of presbylarynx in older women.
