# Supplementary material for: Reference values for electroglottographic data of elderly women
Source: Codas. 2023 Nov 10;35(5):e20210244. doi: 10.1590/2317-1782/20232021244en (PMC10688290; doi:10.1590/2317-1782/20232021244en)
Supplement: Translation

# Translation: Valores de referência para dados eletroglotográficos de idosas

**DOI:** 10.1590/2317-1782/20232021244pt

**Authors:** Bárbara de Faria Morais Nogueira, Ana Cristina Côrtes Gama, Flávio Barbosa Nunes, Adriane Mesquita de Medeiros

**Affiliations:** 1 Departamento de Fonoaudiologia, Faculdade de Medicina, Universidade Federal de Minas Gerais - UFMG, Belo Horizonte (MG), Brasil.; 2 Programa de Pós-graduação em Ciências Fonoaudiológicas, Universidade Federal de Minas Gerais - UFMG - Belo Horizonte (MG), Brasil.; 3 Departamento de Oftalmologia e Otorrinolaringologia, Faculdade de Medicina, Universidade Federal de Minas Gerais - UFMG, Belo Horizonte (MG), Brasil.

**Keywords:** Idoso, Voz, Envelhecimento, Fonação, Mulheres

## Abstract

**Objetivo:**

Apresentar valores de referência para os dados de frequência fundamental e quociente de fase fechada extraído da eletroglotografia em idosas e verificar se diferem com o resultado do exame laríngeo.

**Método:**

Estudo observacional analítico transversal, realizado com 73 idosas na faixa etária de 60 a 84 anos. Foram realizadas avaliações eletroglotográficas e laríngeas. O material de voz coletado de cada participante foi a emissão sustentada da vogal /a/, emitida em frequência e intensidade habituais, no registro modal, de modo prolongado. As medidas eletroglotográficas extraídas foram: Frequência fundamental (F0) e quociente de contato (CQ) extraídos da eletroglotografia (EGG). Foi realizada análise descritiva dos dados por meio da análise das medidas de tendência central e de dispersão das variáveis contínuas. Para estabelecer se as variáveis contínuas do EGG diferem de acordo com o diagnóstico laríngeo, foram utilizados os testes t-Student (teste paramétrico) e Mann-Whitney (não paramétrico). Para todos os testes foi considerado o nível de significância de 5%.

**Resultados:**

A maioria das participantes não possui alterações laríngeas, 27,4% têm presbilaringe. A média das medidas eletoglotográficas foram: 187,24 Hz de F0 e 48,49% de QC. Não houve significância estatística ao comparar o exame laríngeo segundo as medidas do EGG.

**Conclusão:**

Mulheres idosas apresentam valores médios de F0 EGG de 187,24 Hz. O valor médio obtido no QC foi de 48,49%. As medidas eletroglotográficas de F0 e QC não se modificaram com a presença de presbilaringe em idosas.

## INTRODUÇÃO

O envelhecimento natural da voz possui início e desenvolvimento que dependem da saúde física, psíquica, da história de vida do indivíduo além de fatores constitucionais, raciais, hereditários, alimentares, sociais e ambientais^(1)^. Os primeiros sinais do envelhecimento ocorrem por volta dos 60 anos de idade, idade cronológica que considera o indivíduo como idoso segundo a Organização Mundial da Saúde (OMS)^(2)^. Dentre as alterações vocais encontradas nesse período denominada presbifonia pode-se citar a redução da intensidade vocal, a dificuldade no controle muscular da laringe e a limitação respiratória^(3-5)^. A alteração da produção vocal decorrente desse processo pode estar associada ou não à presbilaringe^(5)^, que é o envelhecimento natural da laringe decorrente do avanço da idade.

No exame de videolaringoscopia de idosos com alterações sugestivas de presbilaringe é possível verificar algumas modificações na laringe como: posição mais baixa no pescoço, incompetência glótica, atrofia e redução da espessura das pregas vocais, arqueamento e irregularidade na vibração das pregas vocais, medialização compensatória das bandas ventriculares, ossificação e calcificação das cartilagens, fragilidade dos vasos sanguíneos com tendência à hemorragia submucosa e proeminência do processo vocal^(4-6)^. No processo de envelhecimento normal, a atrofia das pregas vocais está presente em até 60% de indivíduos após os 60 anos apresentando evidências de insuficiência glótica^(4)^.

O diagnóstico da presbifonia é realizado por meio de exclusão^(5)^. Sendo assim, é fundamental realizar uma anamnese detalhada, analisar a presença de patologias na laringe, comorbidades, doenças sistêmicas, uso de medicamentos e tratamentos que possam estar interferindo na qualidade vocal. Deve-se considerar a multicausalidade da disfonia e estabelecer o raciocínio clínico a partir do que é esperado para o diagnóstico de presbifonia.

A realização do exame de eletroglotografia - EGG contribui para maior compreensão do funcionamento da laringe, uma vez que este avalia a fase fechada do ciclo glótico. Sendo assim, é possível verificar possíveis alterações na laringe. A EGG é um método objetivo, não invasivo, de simples medida, utilizada para estimar a movimentação das pregas vocais durante a fonação^(7)^. Esse método fornece uma medida indireta do contato das pregas vocais na fonação (quociente de contato das pregas vocais-QC) que mostra a proporção de tempo em que a glote permanece fechada em relação ao total de tempo do ciclo glótico^(8)^. Esta medida é obtida a partir da variação da corrente elétrica, que muda à medida que as pregas vocais aumentam e diminuem seu grau de contato^(9)^.

Com o envelhecimento espera-se que haja mudanças nas medidas eletroglotográficas devido às modificações estruturais esperadas das pregas vocais com o avanço da idade. O QC pode ser afetado por alterações do fechamento glótico das pregas vocais e a frequência fundamental eletroglotográfica (F0 EGG) pelas mudanças anatômicas e funcionais observadas na laringe dos idosos^(8,9)^. Compreender as características eletroglotográficas das medidas de QC e de F0 EGG entre idosos com e sem presbilaringe é importante para subsidiar a avaliação deste grupo etário, considerando que as diferenças anatômicas e funcionais da laringe dos idosos podem impactar nos valores destas medidas. A literatura apresenta valores de referência de medidas EGG para a população adulta^(10-14)^, infantil^(15)^, e idosa^(8,16,17)^, mas ainda são poucos os estudos que analisam o comportamento das medidas EGG em falantes do português brasileiro^(11-14)^.

Aspectos relacionados à tarefa de fala^(8)^ e as variações linguísticas decorrentes de padrões culturais da língua^(12)^ podem interferir nos valores das medidas EGG. Portanto, analisar os parâmetros EGG na população brasileira idosa pode auxiliar na avaliação da função vocal deste grupo etário e, contribuir para a utilização de medidas objetivas da função vocal no manejo multidimensional dos distúrbios vocais. O presente estudo buscou responder às seguintes questões: Quais os valores das medidas da EGG em idosas? Os resultados se diferem na presença de presbilaringe? Supõe-se que as modificações anatomofuncionais das pregas vocais decorrentes do envelhecimento^(4-6)^ diminuam os valores QC, caracterizando um menor tempo de fase fechada do ciclo glótico, e elevem a F0 EGG.

Desse modo, o objetivo da presente pesquisa foi apresentar os valores de referência para os dados de F0 e QC extraídos do EGG em idosas, verificar se os resultados diferem do resultado da avaliação laríngea.

## MÉTODO

Trata-se de um estudo observacional analítico transversal, aprovado pelo Comitê de Ética em Pesquisa - COEP, sob número de parecer 2.648.174.

Todas as participantes do estudo assinaram o Termo de Consentimento Livre e Esclarecido - TCLE, e realizaram os seguintes procedimentos para esta pesquisa, em ordem de ocorrência: questionário com dados sobre idade, escolaridade, situação de aposentadoria, realização de tratamento fonoaudiológico pregresso e avaliação eletroglotográfica realizada por uma das pesquisadoras para extração dos dados de F0 EGG e do QC. Após a gravação, as participantes foram agendadas para avaliação otorrinolaringológica (ORL), em até 15 dias. A avaliação ORL constou de exame de laringoscopia realizado por um único médico e de aplicação da versão traduzida do protocolo *Reflux Finding Score* (RFS)^(18)^.

Os critérios de inclusão foram: ser mulher com idade igual ou superior a 60 anos capazes de responderem perguntas relacionadas à idade e não ter realizado tratamento fonoaudiológico para o problema de voz nos últimos 12 meses, executar com facilidade as tarefas solicitadas para a avaliação laríngea e vocal. Os critérios de exclusão para esse estudo foram: presença de diagnóstico otorrinolaringológico com alteração laríngea que não caracterize a presbilaringe, diagnóstico de refluxo laringofaríngeo (RLF) segundo o protocolo RFS e a não realização da avaliação laríngea.

A pesquisa ocorreu no Observatório de Saúde Funcional em Fonoaudiologia - OSF de uma universidade pública onde foram realizados os registros das gravações eletroglotográficas das vozes. A videolaringoscopia foi realizada por um médico otorrinolaringologista no Hospital pertencente à mesma universidade.

A amostragem das participantes foi por conveniência. A pesquisa foi divulgada por meio de chamada à comunidade com informativo afixado nos arredores e corredores da universidade e do hospital universitário. O informativo continha o objetivo do estudo, os critérios de inclusão e exclusão, o e-mail e o telefone de contato da pesquisadora. Foi realizada também abordagem face a face da equipe de pesquisa nas proximidades do hospital universitário para divulgação e convite às idosas que estavam nas redondezas. As idosas que demonstraram interesse em participar da pesquisa forneceram seu nome e telefone de contato para posterior contato e agendamento de horário para coleta de dados. Ressalta-se que nenhuma idosa entrou em contato por meio do informativo divulgado à comunidade. Todas as participantes desta pesquisa foram recrutadas por meio da abordagem face a face e, agendadas posteriormente, pela pesquisadora que entrou em contato por telefone.

Ao todo foram convidadas 158 idosas. Todas foram agendadas e 95 compareceram ao OSF para realização da coleta de dados. Dessas, apenas uma idosa não realizou o exame de EEG e 21 não compareceram no dia agendado para realização do exame laríngeo. Sendo assim, foram excluídas da pesquisa 22 participantes. Totalizando, dessa forma, 73 idosas participantes do presente estudo.

O material de voz coletado de cada participante foi a emissão sustentada da vogal /a/, emitida em frequência e intensidade habituais, no registro modal, de modo prolongado. Todas as participantes foram orientadas a realizar a emissão em frequências e intensidades confortáveis, e o fonoaudiólogo controlou, de forma perceptivo-auditiva, se as emissões eram habituais. As participantes foram posicionadas sentadas confortavelmente de maneira a minimizar os seus movimentos durante a captação do traçado eletroglotográfico. A captação da onda pelo traçado eletroglotográfico foi verificada por meio da colocação simétrica de dois eletrodos, limpos e despolarizados com solução salina, de forma superficial, nas lâminas das cartilagens tireoideas ao nível das pregas vocais, conectados e diretamente digitalizados no computador. A coleta foi realizada em ambiente silencioso com um nível de ruído ambiental inferior a 50 dB NPS verificado através do medidor de nível de pressão sonora da marca Radio Shack® utilizando-se o programa CSL da Kay PentaxTM, model 6103, Lincoln Park NJ USA - módulo Electroglottography instalado no computador da marca Dell®, modelo Optiplex GX260, com placa de som profissional marca Direct Sound®. Não foi realizado o controle objetivo da intensidade durante a emissão da vogal sustentada.

As medidas eletroglotográficas extraídas foram: F0 EGG que se refere ao número de ciclos glóticos produzidos em um intervalo de tempo de um segundo. A unidade de medida é o hertz (Hz). E o QC: corresponde à porcentagem (%) de cada ciclo glótico em que as pregas vocais ficaram fechadas.

Após a coleta das medidas eletroglotográficas as idosas foram submetidas à avaliação da laringe realizada por um médico otorrinolaringologista por meio da videolaringoscopia, composta por telescope 70°, marca Storz®, fonte de luz xênon de 300 Watts, marca Storz®, microcâmera telecam DX, marca Storz® em um intervalo máximo entre as avaliações de 15 dias. Foi solicitado que todas as participantes emitissem a vogal sustentada /i/ durante o período mínimo de dois segundos na mesma frequência habitual. Todas as informações do exame foram registradas em um laudo médico que continha também a versão traduzida do RFS^(18)^. Todas as participantes receberam o laudo médico ao final do exame. A pesquisadora também recebeu uma cópia do exame de cada participante. No RFS são avaliados: edema subglótico (ausente/presente), obliteração dos ventrículos (parcial/completa), eritema ou hiperemia (somente nas aritenoides/difusa), edema de pregas vocais (leve/moderado/grave/polipoide), edema laríngeo difuso, hipertrofia da região interaritenóidea (leve/moderado/grave/obstrutivo), granuloma/tecido de granulação (ausente/presente), muco endolaríngeo espesso (ausente/presente). Para chegar à pontuação final foi realizado o somatório de todos os itens do protocolo. Quando o resultado for superior a 7 é indicativo de diagnóstico de RLF^(18)^.

O diagnóstico otorrinolaringológico de presbilaringe foi definido a partir da presença de: 1) fenda glótica; 2) atrofia e redução da espessura das pregas vocais; e 3) proeminência do processo vocal^(5)^. O resultado do exame laríngeo foi separado em dois grupos: sem alterações laríngeas e presença de presbilaringe. Os critérios para compor o grupo sem alterações laríngeas foram: não apresentar alteração na mucosa, ter coaptação glótica completa das pregas vocais e não preencher o critério adotado pelo RFS para o diagnóstico de RLF. Para compor o grupo de presbilaringe as idosas deveriam apresentar os três sinais laríngeos acima descritos^(5)^, e não preencher o critério adotado pelo RFS para o diagnóstico de RLF.

Após a coleta, as informações foram digitalizadas em um banco de dados no programa Microsoft Office Excel. Todas as análises foram realizadas no software IBM - SPSS Statistic Base, versão 25.0. A análise descritiva dos dados foi realizada por meio da análise das medidas de tendência central e de dispersão das variáveis contínuas e o teste de normalidade usado foi o Shapiro Wilk. A comparação dos grupos com e sem alteração no exame laríngeo para as medidas do EGG foi analisada com os testes t-Student (teste paramétrico) e Mann-Whitney (não paramétrico). Para todos os testes foi considerado o nível de significância de 5%.

## RESULTADOS

A pesquisa foi realizada com 73 idosas com média de idade de 69 anos (desvio padrão=5,75). A maior parte das participantes possui ensino médio (31,5%) e a maioria é aposentada (90,4%). A média do QC das participantes foi de 48,49 (desvio padrão=8,15) e da F0 187,24 Hz (desvio padrão=31,76). Com relação à avaliação laríngea 72,6% das participantes não possuem alterações laríngeas e 27,4% possuem presbilaringe. A presbilaringe foi descrita no exame laríngeo como presença de arqueamento de pregas vocais com fenda fusiforme. Nenhuma das participantes do estudo apresentou diagnóstico de RLF, de acordo com o protocolo utilizado no exame de laringoscopia. As medidas de tendência central da idade e das medidas eletroglotográficas encontram-se na Tabela 1.

**Tabela 1:** Medidas descritivas das variáveis: idade, quociente de contato e frequência fundamental da Eletroglotografia EGG

**Variáveis**	**N**	**Média**	**D.P.**	**Mediana**	**Mínimo**	**1º Q**	**3º Q**	**Máximo**
Idade (anos)	73	69,22	5,75	69	60	65	73	84
Quociente de contato	73	48,49	8,15	48,16	6,9	43,81	52,62	68,7
Frequência fundamental	73	187,24	31,76	186,15	127,37	166,49	203,98	264,65

**Legenda:** N = número de indivíduos; D.P.= desvio padrão; Q = quartil

Na Tabela 2 foi realizada comparação dos grupos sem alteração laríngea e com presbilaringe quanto às medidas do EGG (QC e F0) na qual não se observou diferença estatística entre os grupos.

**Tabela 2:** Comparação do quociente de contato e da frequência fundamental da Eletroglotografia (n=73), entre os grupos

**Variáveis**	**Grupos avaliados**		
	Sem alteração laríngea	Presbilaringe	**Valor-p**
	(n=53)	(n=20)	
**Frequência fundamental** 1 **(em Hz)**			
Média	187,31	187,04	0,9745
Mediana	186,76	185,70	
**Quociente de contato** 2 **(em %)**			
Média	47,41	49,39	0,3105
Mediana	47,87	49,57	

1 Teste t-Studen, t;

2 Teste Mann-Whitney

**Legenda:** Hz=Hertz

## DISCUSSÃO

As medidas eletroglotográficas de F0 e QC das idosas do presente estudo não diferenciaram com os diagnósticos laríngeos (sem alteração e com presença de presbilaringe). A baixa prevalência (27,4%) de presbilaringe encontrada pode ter ocorrido em decorrência da idade desse estudo. Metade das idosas tinham menos de 69 anos e somente 25% apresentaram idade entre 73 e 84 anos. Estudo com 104 idosas saudáveis com idade acima de 65 anos encontrou 45,2% com presença de presbilaringe (fenda fusiforme)^(17)^.

São escassos os estudos que definem os parâmetros de normalidade das medidas eletrogrotográficas em idosas^(8,16,17)^. Os resultados desta pesquisa mostram valores de F0 EGG em idosas sem alteração laríngea de 187,31 Hz, e de 187,04 Hz de idosas com presbilaringe. Tais resultados são similares aos valores apontados pela literatura em mulheres idosas de 186,05 Hz^(8)^ e de 211,06 Hz^(17)^, respectivamente.

Considerando a população de mulheres adultas, valores de média da F0 EGG foram de 226,91 Hz em mulheres cantoras^(11)^, 204,87 Hz em mulheres adultas sem alteração vocal^(12)^, assim como de 211,69 Hz em mulheres sem alteração laríngea^(14)^. Os resultados sugerem que na população adulta feminina, os valores de F0 EGG tendem a ser levemente superiores aos valores encontrados em mulheres idosas.

Observamos que, na população idosa, a presença de presbilaringe não impacta os valores de F0 EGG, como observado pela literatura^(17)^, sugerindo que a presença dos sinais laríngeos de atrofia da mucosa e redução da espessura das pregas vocais^(5)^, não impactam no parâmetro EGG da F0, ou seja, o fator idade é mais importante para definir a F0 que as alterações da prega vocal já visíveis ao exame laríngeo. É lícito supor que o envelhecimento gera modificações nas estruturas das pregas vocais^(5)^ que impactam na F0 EGG, independentemente destas alterações laríngeas já serem identificadas visualmente na avaliação laringológica.

Vale ressaltar que a extração da medida da F0 como medida eletroglotográfica difere da obtenção por meio da análise acústica da onda sonora. A F0 na EGG é de mais fácil extração do que na análise acústica, pois representa ciclos mais nítidos^(1)^. Na análise acústica obtêm-se sinais de voz por meio de alguns parâmetros acústicos como a F0^(19)^ enquanto que na EGG estima-se a área de contato entre as pregas vocais durante a produção vocal, onde também é possível extrair a medida da F0^(1)^.

Os dados eletroglotográficos permitem analisar o contato das pregas vocais durante o ciclo glótico, sendo utilizados para avaliar a função vocal^(7,10,13)^. Os resultados apresentados podem contribuir para análise das medidas eletroglotográficas de mulheres idosas extraídas por meio do programa CSL da Kay Pentax™. Diferença dos programas e dos critérios de avaliação podem alterar os resultados dos parâmetros eletroglotográficos^(8)^, devendo-se ter cautela na comparação dos resultados das pesquisas.

Quanto ao QC, a média de 48,49%, verificada no presente estudo, encontra-se dentro dos padrões de normalidade (intervalo de 40-60%) descrito pela literatura^(10)^, sugerindo que o envelhecimento não interfere neste parâmetro da EGG. Nesta pesquisa, as mulheres idosas sem alteração laríngea apresentaram valores de QC de 47,41%, e as idosas com presbilaringe, valores de QC de 49,39, sem diferenças estatisticamente significantes entre os grupos. Dentre os achados que caracterizam a prebilaringe, a insuficiência glótica tem se mostrado predominante^(4)^. Com o avanço da idade almeja-se que ocorra redução na duração do fechamento glótico nas mulheres^(16)^, porém a presença de fenda glótica, observada em todas as idosas do grupo de presbilaringe, não interferiu nos valores de QC. Resultado do QC de 96 idosas com e sem presbilaringe encontrou a média de 47,6%^(17)^.Os valores de QC também foram imprecisos para identificação da presença de fenda glótica em idosas, confirmando os achados desta pesquisa. Os autores discutem sobre a possível interferência do tom e da intensidade da voz na avaliação do QC^(17)^.

Os resultados da presente pesquisa permitem sugerir que o envelhecimento impacta, quando comparados com a população adulta, nos valores de F0 EGG, com leve redução dos valores desta medida, e não interfere no tempo do fechamento glótico, e consequentemente, nos valores de QC, que se encontram próximos aos da população de mulheres adultas. Tais resultados são confirmados mesmo na presença da presbilaringe. É lícito supor, que as modificações anatômicas e funcionais observadas na população idosa, e principalmente nos casos de presbilaringe, não são suficientes para modificar as medidas eletroglotográficas desta população, sugerindo que as modificações anatomofuncionais decorrentes do envelhecimento diminuem a velocidade de vibração das pregas vocais, com redução da F0 EGG, mas não modificam o tempo da fase fechada do ciclo glótico, sem impacto nos valores do QC EGG. A normalização e experiência prévia com o EGG é aconselhável com maior controle na captação da emissão vocal quanto ao tom e intensidade da voz.

Como limitação do estudo podemos destacar o tamanho da amostra reduzido, principalmente do grupo de presbilaringe, e a amostragem por conveniência, o que impede a generalização dos resultados. Além do mais a restrição de estudos científicos com resultados eletroglotográficos na população idosa e a falta de normatização das medidas dificultam a interpretação dos resultados.

A presente pesquisa contribuiu para a identificação de valores de referências para os dados de F0 e QC extraídos da EGG em condições laríngeas distintas na população idosa, auxiliando no processo da avaliação multidimensional da voz da população de mulheres idosas. São necessários dados normativos para se analisar a conformidade, a confiança e a variabilidade das medições de parâmetros vocais entre pessoas de diversas faixas etárias e sexos. Sugere-se a investigação sobre a diferença dos resultados do CQ com características laríngeas (de presbilaringe e laringes sem alterações) entre as idosas considerando a faixa etária.

Torna-se também necessário a realização de novos estudos em EEG com idosos de ambos os sexos a fim de averiguar a possibilidade da padronização desse instrumento nessa população, e sua colaboração no diagnóstico da presbilaringe e seus impactos vocais.

## CONCLUSÃO

Mulheres idosas apresentam valores médios de F0 EGG de 187,24 Hz. O valor médio obtido no QC foi de 48,49%. As medidas eletroglotográficas de F0 e QC não se modificaram com a presença de presbilaringe em idosas.
